# Pharmacological Modulation of Catecholaminergic Brain Injury by Dapagliflozin and Its Nanovesicular Formulation in an Isoproterenol Model

**DOI:** 10.1002/jbt.71134

**Published:** 2026-10-18

**Authors:** Faisal Ashraf Bhat, Syed Ehtaishamul Haque, Mohammed Aqil, Mohd Akhtar, Abul Kalam Najmi

**Affiliations:** ^1^ Department of Pharmacology School of Pharmaceutical Education and Research, Jamia Hamdard (SPER) New Delhi Delhi India; ^2^ Department of Pharmaceutics School of Pharmaceutical Education and Research, Jamia Hamdard New Delhi Delhi India

**Keywords:** catecholamine, catecholaminergic, dapagliflozin, drug delivery, hippocampal formation, medicine, neuroprotection, neurotrophic factors, neurotrophin, pharmacology

## Abstract

Sustained β‐adrenergic stimulation represents a pharmacologically relevant stressor capable of inducing systemic oxidative and inflammatory injury that extends to the central nervous system. Isoproterenol (ISO), a synthetic catecholamine, is widely used to model catecholaminergic injury in experimental settings. Dapagliflozin, a sodium–glucose cotransporter‐2 (SGLT2) inhibitor, exhibits pleiotropic antioxidant and anti‐inflammatory properties; however, its effects on catecholamine‐induced brain injury remain poorly defined. In the present study, we investigated the pharmacological effects of dapagliflozin and a bile salt–stabilized dapagliflozin‐loaded nanovesicular formulation in an ISO‐induced model of catecholaminergic brain injury in rats. ISO administration resulted in marked oxidative imbalance, neuroinflammatory activation, behavioral dysfunction, and structural alterations in hippocampal and cortical regions. These changes were accompanied by reduced expression of stress‐responsive and neurotrophic proteins, including SIRT6, TRAP1, and BDNF, along with enhanced astroglial activation as indicated by increased GFAP immunoreactivity. Treatment with dapagliflozin attenuated these molecular, cellular, and functional alterations, while the nanovesicular formulation produced a more pronounced improvement of redox balance, suppression of inflammatory responses, normalization of protein expression, and preservation of neuronal architecture and behavior. Collectively, these findings demonstrate that dapagliflozin mitigates catecholaminergic brain injury through attenuation of oxidative and inflammatory stress, with enhanced efficacy achieved via formulation‐assisted drug delivery, without implying a distinct pharmacodynamic mechanism.

## Graphical abstract

Bile salt–stabilized nanovesicular dapagliflozin attenuated oxidative stress, neuroinflammation, and neuronal injury while preserving stress‐response proteins and improving behavioral outcomes in an experimental model of isoproterenol‐induced catecholaminergic brain injury.

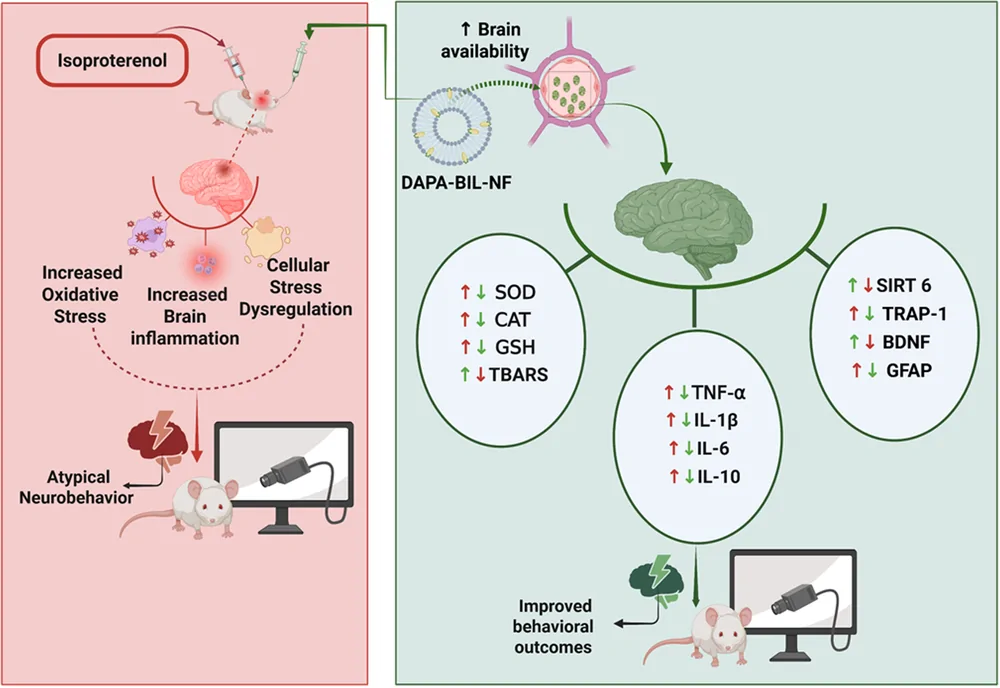

## 1 Introduction

Isoproterenol (ISO) is a synthetic catecholamine with potent β‐adrenergic agonistic activity, and its administration at higher doses reliably induces myocardial ischemia, establishing its utility as a standard preclinical model of catecholamine‐mediated cardiac injury [1]. Beyond its well‐characterized cardiovascular effects, accumulating evidence indicates that ISO‐induced catecholaminergic toxicity is not confined to peripheral tissues but also extends to the central nervous system [2]. Persistent sympathetic activation during myocardial ischemia and infarction not only drives maladaptive cardiac remodeling but is increasingly recognized to impair neural circuits involved in cognition, affective regulation, and stress responsiveness, underscoring the relevance of coordinated cardiovascular–neural interactions under conditions of systemic catecholamine overload stress [3].

Experimental studies have demonstrated that high‐dose ISO exposure provokes pronounced oxidative and inflammatory injury in the brain, potentially mediated through nicotinamide adenine dinucleotide phosphate (NADPH) oxidase (NOX)‐dependent mechanisms [4]. Sustained β‐adrenergic stimulation has been shown to activate NOX‐driven oxidative pathways in astrocytes, leading to endoplasmic reticulum stress and nuclear factor‐κB (NF‐κB) signaling, thereby promoting neuroinflammation and disruption of cerebral homeostasis [2]. Prolonged oxidative and inflammatory insults further compromise cellular quality‐control systems and proteostatic balance, increasing neuronal vulnerability and predisposing neural tissue to functional impairment [5].

Dapagliflozin (DAPA), a sodium–glucose cotransporter‐2 (SGLT2) inhibitor, is well recognized for its cardioprotective benefits; however, emerging evidence suggests that its pleiotropic antioxidant and anti‐inflammatory properties may also mitigate neural injury [6]. Despite extensive investigation of dapagliflozin in cardiovascular and metabolic disorders, its potential role in attenuating isoproterenol‐induced brain toxicity remains largely unexplored, representing an important gap in the current literature. In this context, a bile salt–stabilized dapagliflozin‐loaded nanovesicular formulation has previously been shown to enhance the pharmacological efficacy of dapagliflozin in cardiovascular settings, although its impact on central neuroinflammatory and oxidative injury has not been evaluated.

Accordingly, the present study was designed to investigate the protective effects of dapagliflozin suspension and its nanovesicular formulation in an ISO‐induced model of catecholamine‐mediated neurotoxicity. Neurobehavioral performance was assessed alongside the evaluation of oxidative stress markers, inflammatory cytokines, histopathological alterations, and immunohistochemical expression of key stress‐responsive and neurotrophic proteins, including SIRT6, TRAP1, BDNF, and GFAP, in the hippocampus and cortex. Through this integrated approach, we sought to determine whether formulation‐assisted SGLT2 inhibition could attenuate oxidative and inflammatory brain injury and improve functional outcomes under sustained adrenergic stress.

## 2 Materials and Methods

### 2.1 Drugs & Chemicals

Dapagliflozin (purity > 98%) was kindly provided as a gift sample by Sun Pharmaceutical Industries Ltd. (India). Isoproterenol hydrochloride was purchased from Sigma‐Aldrich (St. Louis, MO, USA). Brain levels of tumor necrosis factor‐α (TNF‐α; EliKine Rat ELISA Kit, Cat. No. KTE9007), interleukin‐6 (IL‐6; Cat. No. KTE9004), interleukin‐1β (IL‐1β; Cat. No. KTE9001), and interleukin‐10 (IL‐10; Cat. No. KTE9005) were quantified using commercially available quantitative sandwich ELISA kits (Abbkine Inc., Atlanta, GA, USA; supplied by Amplicon Biotech, India). The ELISA kits exhibited analyte‐specific detection ranges and sensitivities, with intra‐ and inter‐assay coefficients of variation of < 10% and < 12%, respectively, according to the manufacturer's specifications. Primary antibodies used for immunohistochemistry included rabbit recombinant monoclonal anti‐SIRT6 (ABclonal Technology, Wuhan, China; Cat. No. A28245; 1:200), rabbit polyclonal anti‐TRAP1 (ABclonal Technology; Cat. No. A2748; 1:100), rabbit polyclonal anti‐BDNF (Abcam, Cambridge, UK; Cat. No. ab226843; 1:100), and rabbit monoclonal anti‐GFAP (ABclonal Technology; Cat. No. A19058; Clone ARC0206; 1:200). All other chemicals and reagents used in the study were of analytical grade.

### 2.2 Experimental Animals and Study Design

Male Wistar albino rats (170–190 g) were procured from the Central Animal House, Jamia Hamdard, New Delhi, India. Animals were housed under standard laboratory conditions (temperature 22°C ± 2°C, relative humidity 55% ± 5%, 12 h light/dark cycle) with free access to standard pellet diet and water. All experimental procedures were approved by the Institutional Animal Ethics Committee (IAEC approval no. 1644) and were conducted in accordance with CCSEA (Committee for Control and Supervision of Experiments on Animals) guidelines.

After acclimatization, animals were randomly allocated into six groups (*n* = 6 per group). The dosing schedule and experimental timeline are illustrated in Figure 1. Isoproterenol was administered at a dose of 100 mg/kg/bw via subcutaneous injection at 24‐h intervals for 2 consecutive days (Days 13 and 14) to induce catecholaminergic stress [1]. This dose was selected based on previous studies demonstrating that high‐dose isoproterenol reliably induces systemic oxidative stress, neuroinflammation, behavioral impairment and neuronal injury, thereby serving as a validated model of catecholaminergic brain injury [1, 7]. Dapagliflozin suspension and the dapagliflozin‐loaded nanovesicular formulation (DAPA‐BIL‐NF) were administered orally at an equivalent dapagliflozin dose of 1 mg/kg/day for 14 days [6]. The dose and treatment duration were selected based on previous reports demonstrating antioxidant, anti‐inflammatory, and neuroprotective efficacy of dapagliflozin in experimental neurological injury models [8, 9].

**FIGURE 1:**
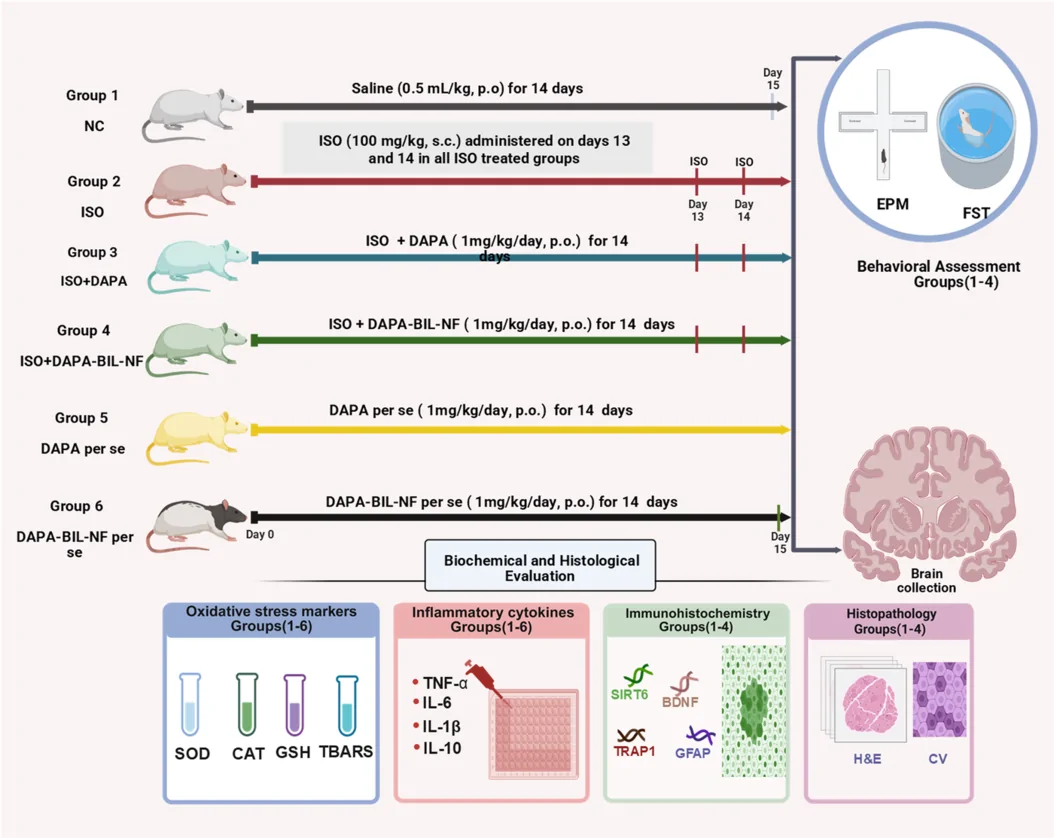
Graphical representation of treatment schedule, duration, and the targeted biochemical and histopathological parameters.

On day 15th day, animals were euthanized by gradual‐fill CO_2_ inhalation in accordance with the IAEC‐approved protocol under the Committee for Control and Supervision of Experiments on Animals (CCSEA) guidelines. Death was confirmed prior to brain tissue collection. Blood samples were collected, and brains were rapidly excised, rinsed in ice‐cold saline, and placed on ice. The frontal cortex and hippocampus were dissected. Tissue samples were either fixed in 10% neutral buffered formalin for histopathological and immunohistochemical analyses or snap‐frozen at −20°C for biochemical estimations.

### 2.3 Preparation and Administration of Dapagliflozin Nanovesicular Formulation

The optimized DAPA‐BIL‐NF formulation, previously characterized by our group, exhibited a mean vesicle size of 120.9 ± 3.21 nm, a zeta potential of −41.69 ± 0.023 mV, and an entrapment efficiency of 92.8% ± 5.46%. Transmission electron microscopy demonstrated predominantly spherical vesicles with uniform morphology, while FTIR and XRD analyses confirmed successful drug incorporation without evidence of chemical incompatibility. In addition, the formulation exhibited sustained drug release over 24 h and enhanced intestinal penetration in ex vivo studies. These physicochemical characteristics supported its selection for the present pharmacological investigation [10].

### 2.4 Behavioral Evaluations

All animals underwent preliminary training for 2–3 days prior to experimentation to ensure acclimatization and stable baseline performance. Behavioral assessments were conducted in a quiet, controlled environment and performed in the order of least to most stressful paradigms, with a minimum interval of 24 h between successive tests to minimize stress‐induced interference [5].

#### 2.4.1 Forced Swim Test (FST)

The Forced Swim Test (FST) was used to evaluate behavioral responses to an acute inescapable stressor by measuring immobility, swimming, and climbing behaviors. Rats were individually placed in a transparent cylindrical container (45 × 15 × 45 cm) filled with water maintained at 25°C ± 1°C. Each animal underwent a 15‐min pre‐exposure session, followed 24 h later by a 5‐min test session. Immobility, swimming, and climbing behaviors were recorded during the test period [11].

#### 2.4.2 Elevated Plus Maze (EPM)

Anxiety‐like behavior was assessed using the Elevated Plus Maze apparatus, consisting of two open arms and two closed arms elevated 50 cm above the floor. Each rat was placed at the center of the maze facing an open arm and allowed to explore freely for 5 min. The number of entries into open and closed arms and the time spent in each arm were recorded [12].

### 2.5 Assessment of Oxidative Stress Markers

Brain tissues were homogenized in appropriate buffer, and the homogenates were used for biochemical analysis. Reduced glutathione (GSH) [13], superoxide dismutase (SOD) [14], and catalase (CAT) [15] activities were estimated using standard spectrophotometric methods. Lipid peroxidation was quantified by measuring thiobarbituric acid reactive substances (TBARS) as an index of oxidative damage (TBARS) [16].

### 2.6 Neuroinflammatory Cytokine Estimation

Levels of pro‐ and anti‐inflammatory cytokines (TNF‐α, IL‐6, IL‐1β, and IL‐10) in brain homogenates were quantified using commercially available ELISA kits according to the manufacturer's instructions. Absorbance was measured using a microplate reader at the specified wavelength, and cytokine concentrations were calculated from standard curves generated for each assay.

### 2.7 Histopathology and Immunohistochemistry

Formalin‐fixed brain tissues from the hippocampus (CA1 region) and cortex were processed, paraffin‐embedded, and sectioned at 5 µm thickness. Sections were deparaffinized in xylene, rehydrated through graded ethanol, and rinsed in distilled water. Hematoxylin and eosin (H&E) and Cresyl violet (CV) staining were performed for histopathological evaluation. For immunohistochemistry, antigen retrieval was carried out using standard procedures. Sections were incubated with primary antibodies overnight at 4°C, followed by appropriate secondary antibodies and visualization using a DAB chromogen. Sections were counterstained, dehydrated, and mounted for microscopic analysis [17].

### 2.8 Statistical Analysis

Data are expressed as mean ± SEM (*n* = 6 per group). The sample size was selected based on previously published studies employing similar experimental models, in which six animals per group were sufficient to detect biologically meaningful treatment‐related differences [18, 19]. Prior to statistical analysis, data normality was assessed using the Shapiro–Wilk test. Since the data were normally distributed, comparisons among groups were performed using one‐way analysis of variance (ANOVA) followed by Tukey's multiple comparison post hoc test using GraphPad Prism version 10.0 (GraphPad Software, San Diego, CA, USA). A value of *p* < 0.05 was considered statistically significant.

## 3 Results

### 3.1 Effects of Dapagliflozin and Its Nanoformulation on Brain Oxidative Stress Markers

Brain tissues from the isoproterenol (ISO) group exhibited a significant reduction in superoxide dismutase (SOD), catalase (CAT), and reduced glutathione (GSH), along with a marked increase in thiobarbituric acid reactive substances (TBARS), compared with the control group (*p* < 0.001). Treatment with dapagliflozin (ISO + DAPA) significantly restored SOD, CAT, and GSH levels and reduced TBARS compared with the ISO group (*p* < 0.05). Notably, animals treated with the dapagliflozin‐loaded bilosomal formulation (ISO + DAPA‐BIL‐NF), a previously optimized and physiochemically characterized bilosomal formulation [10] exhibited a more pronounced improvement in antioxidant enzyme levels and a greater reduction in TBARS compared with ISO alone (*p* < 0.01). In contrast, animals receiving DAPA alone or DAPA‐BIL‐NF alone showed no significant alterations in SOD, CAT, GSH, or TBARS levels compared with the control group (*p* > 0.05), indicating that neither treatment affected basal oxidative homeostasis under physiological conditions (Figure 2).

**FIGURE 2:**
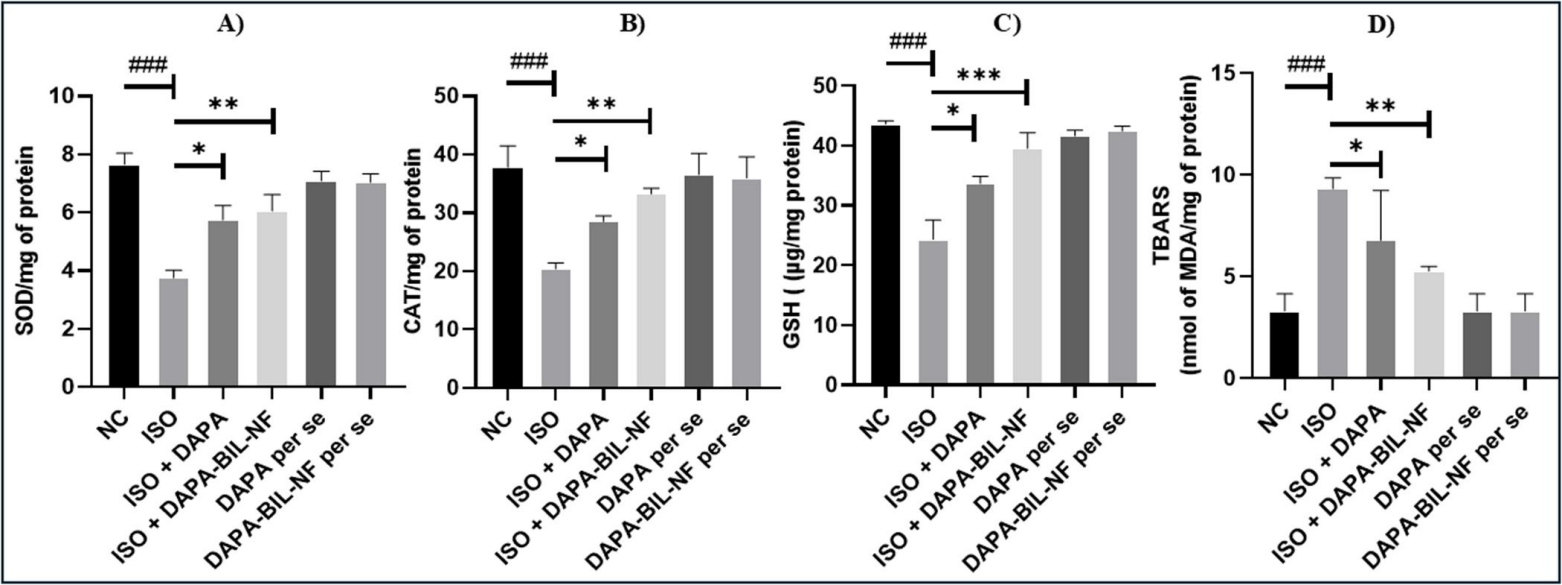
Effect of dapagliflozin (DAPA) and dapagliflozin‐loaded bilosomal nanovesicular formulation (DAPA‐BIL‐NF) on superoxide dismutase (SOD), catalase (CAT), reduced glutathione (GSH), and thiobarbituric acid reactive substances (TBARS) levels in rat brain tissue following isoproterenol (ISO) administration. Data are expressed as mean ± SEM (*n* = 6). Statistical analysis was performed using one‐way ANOVA followed by Tukey's multiple comparison test. ^**###**^ *p* < 0.001 vs. Normal Control (NC); *p < 0.05, **p < 0.01, and ***p < 0.001 vs. the ISO group. DAPA per se and DAPA‐BIL‐NF per se did not differ significantly from the Normal Control group (*p* > 0.05).

### 3.2 Effects of Dapagliflozin Formulations on Brain Inflammatory Markers

The ISO‐treated group showed a significant elevation in the pro‐inflammatory cytokines TNF‐α, IL‐1β, and IL‐6, along with a marked reduction in the anti‐inflammatory cytokine IL‐10, compared with the control group (*p* < 0.001). Administration of dapagliflozin (DAPA) significantly reduced TNF‐α, IL‐1β, and IL‐6 levels and increased IL‐10 levels relative to the ISO group (*p* < 0.05). Notably, treatment with the dapagliflozin‐loaded bilosomal formulation (DAPA‐BIL‐NF) resulted in a more pronounced attenuation of pro‐inflammatory cytokines and increased the IL‐10 levels compared with the ISO group (*p* < 0.001). In contrast, animals receiving DAPA alone or DAPA‐BIL‐NF alone showed no significant changes in TNF‐α, IL‐1β, IL‐6, or IL‐10 levels compared with the control group (*p* > 0.05), indicating that neither treatment altered basal inflammatory status under physiological conditions (Figure 3).

**FIGURE 3:**
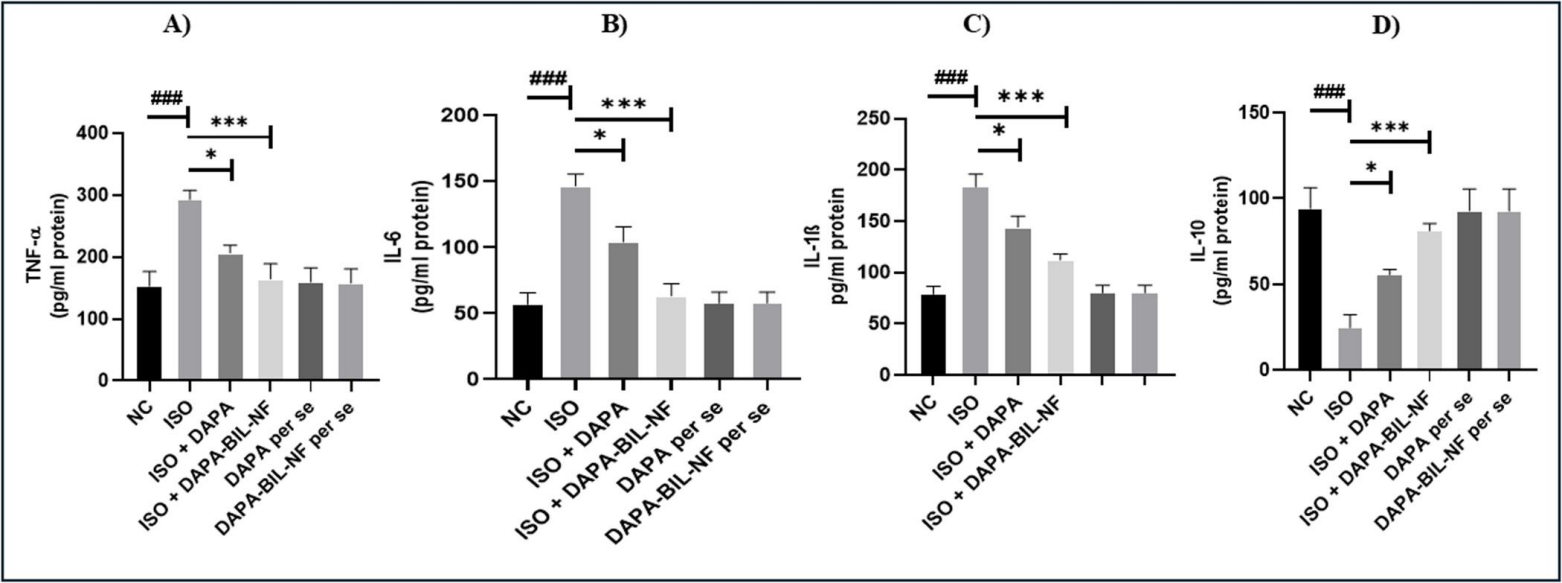
Effect of dapagliflozin (DAPA) and dapagliflozin‐loaded bilosomal nanovesicular formulation (DAPA‐BIL‐NF) on brain levels of tumor necrosis factor‐α (TNF‐α), interleukin‐6 (IL‐6), interleukin‐1β (IL‐1β), and interleukin‐10 (IL‐10) in isoproterenol (ISO)‐treated rats. Data are presented as mean ± SEM (*n* = 6). Statistical analyses were performed using one‐way ANOVA followed by Tukey's multiple comparison post hoc test. ^**###**^ *p* < 0.001 vs. Normal Control (NC); *p < 0.05 and ***p < 0.001 vs. the ISO group. DAPA per se and DAPA‐BIL‐NF per se did not differ significantly from the Normal Control group (*p* > 0.05).

### 3.3 Effects of Dapagliflozin and Its Nano Formulation on Behavioral Outcomes

#### 3.3.1 Forced Swim Test (FST)

ISO‐treated rats exhibited altered behavioral performance, as indicated by increased immobility time and reduced swimming, climbing, and overall performance time compared with the control group (*p* < 0.001). Treatment with dapagliflozin significantly decreased immobility and enhanced active behaviors, including immobility time (*p* < 0.05), climbing time (*p* < 0.05), swimming time (*p* < 0.01), and performance time (*p* < 0.01), relative to the ISO group. Notably, the dapagliflozin‐loaded bilosomal formulation produced a more pronounced performance in FST, with greater normalization of all FST parameters compared with ISO‐treated animals (*p* < 0.001) (Figure 4).

**FIGURE 4:**
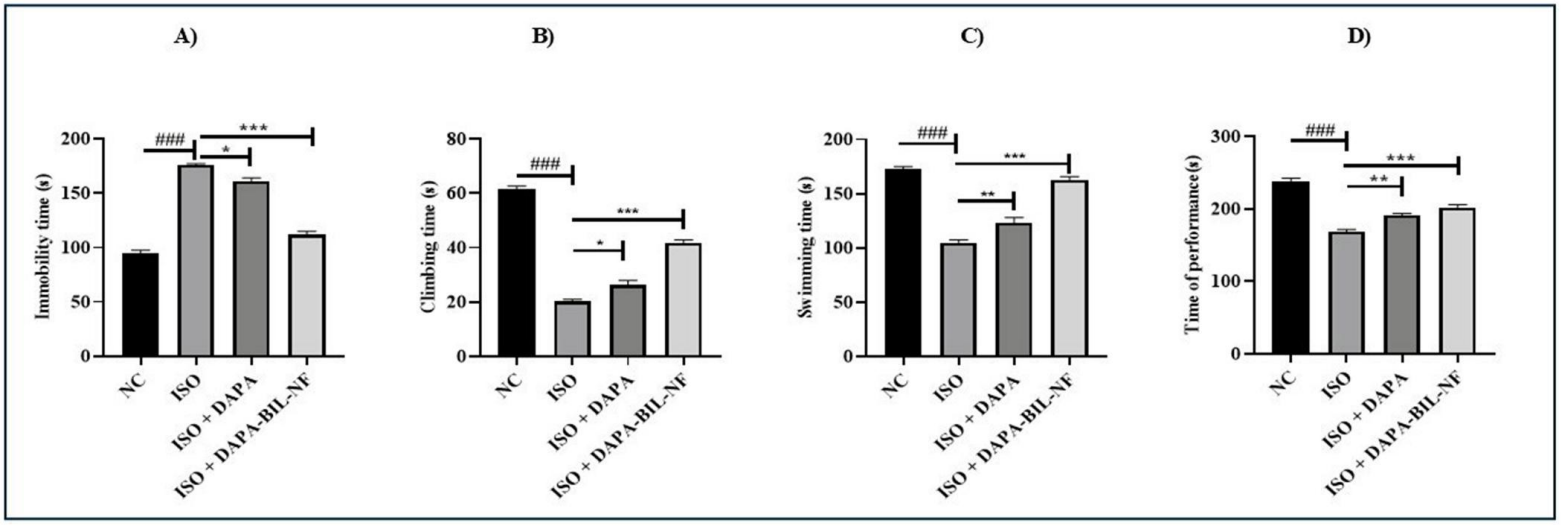
Effect of dapagliflozin (DAPA) and dapagliflozin‐loaded bilosomal nanovesicular formulation (DAPA‐BIL‐NF) on immobility time (A), climbing time (B), swimming time (C), and total performance time (D) in the Forced Swim Test (FST) following isoproterenol (ISO) administration. Data are presented as mean ± SEM (*n* = 6). Statistical analyses were performed using one‐way ANOVA followed by Tukey's multiple comparison post hoc test. ^**###**^ *p* < 0.001 vs. Normal Control (NC); *p < 0.05, **p < 0.01, and ***p < 0.001 vs. the ISO group.

#### 3.3.2 Elevated Plus Maze (EPM)

ISO‐treated rats showed significant anxiety‐like behavior, evidenced by reduced open arm entries and duration and increased closed arm entries and duration compared with the control group (*p* < 0.001). Dapagliflozin treatment significantly improved open arm exploration and reduced closed arm activity relative to the ISO group (*p* < 0.01). Notably, the dapagliflozin‐loaded bilosomal formulation produced a more pronounced anxiolytic effect, with greater normalization of EPM parameters compared with ISO‐treated animals (*p* < 0.001) (Figure 5).

**FIGURE 5:**
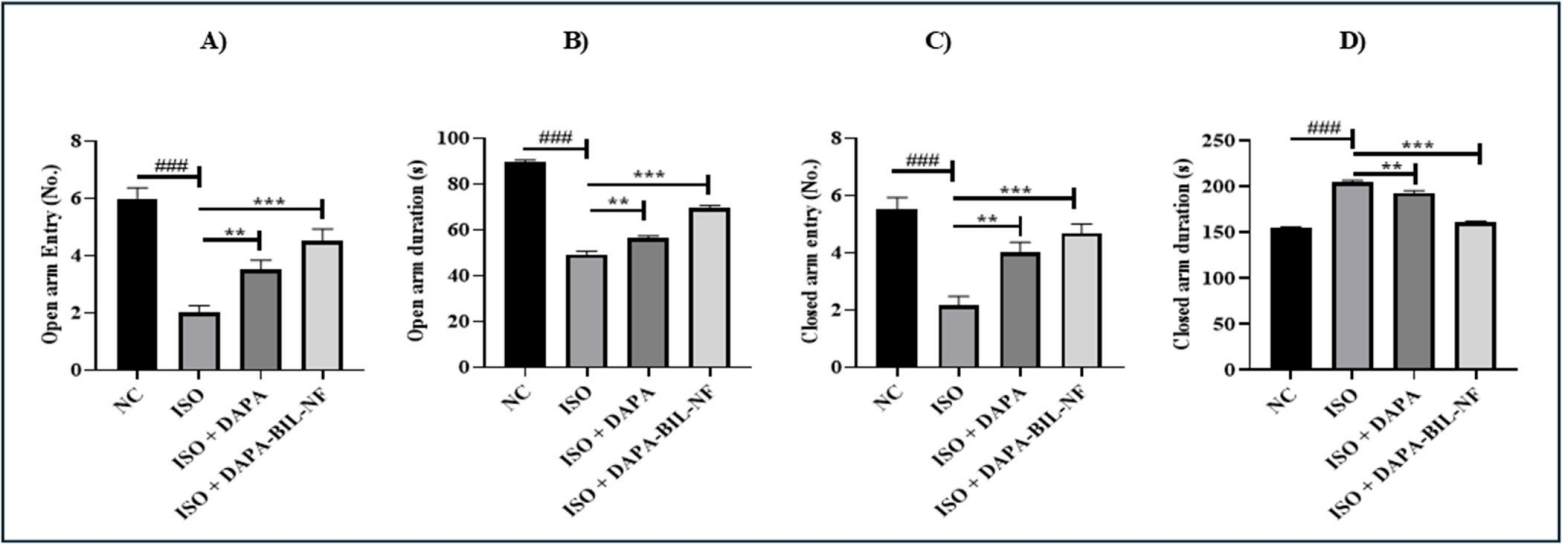
Effect of dapagliflozin (DAPA) and dapagliflozin‐loaded bilosomal nanovesicular formulation (DAPA‐BIL‐NF) on open arm entries (A), open arm duration (B), closed arm entries (C), and closed arm duration (D) in the Elevated Plus Maze (EPM) test following isoproterenol (ISO) administration. Data are presented as mean ± SEM (*n* = 6). Statistical analyses were performed using one‐way ANOVA followed by Tukey's multiple comparison post hoc test. ^###^ *p* < 0.001 vs. Normal Control (NC); *p < 0.05, **p < 0.01, and ***p < 0.001 vs. the ISO group.

### 3.4 Histopathological Evaluation of the Hippocampus (CA1 Region) and Cortex

#### 3.4.1 Hematoxylin and Eosin Staining of the Hippocampal CA1 Region and Cortex

Hematoxylin and eosin staining of the hippocampal CA1 region and cortical areas in control rats revealed normal architecture of the hippocampus and cortex (CA1) region, characterized by healthy cells (green color arrows) in hippocampus and cortex. DAPA treatment resulted in partial preservation of neuronal morphology, whereas DAPA‐BIL‐NF treatment markedly preserved hippocampal and cortical architecture, with reduced neuronal degeneration and improved cellular organization comparable to control tissue (Figure 6).

**FIGURE 6:**
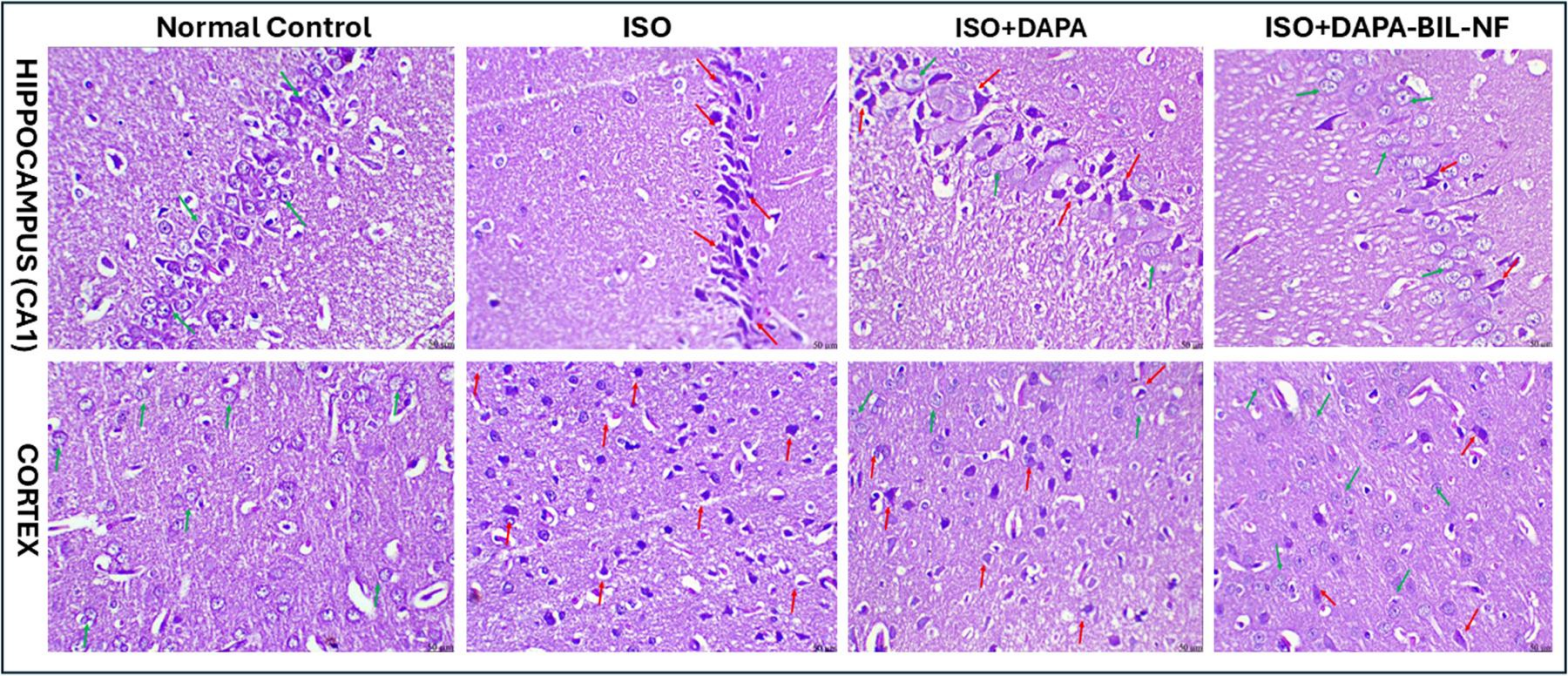
Representative hematoxylin and eosin (H&E)‐stained sections of the hippocampal CA1 region and cortex from control, ISO, ISO + DAPA, and ISO + DAPA‐BIL‐NF groups. Control sections show normal cytoarchitecture with intact neuronal morphology. ISO‐treated rats exhibit neuronal degeneration and disrupted cellular organization. Dapagliflozin treatment shows partial preservation of tissue architecture, while DAPA‐BIL‐NF treatment demonstrates improved preservation of neuronal morphology and structural organization. Scale bar = 50 µm.

#### 3.4.2 Cresyl Violet Staining of the Hippocampal CA1 Region and Cortex

Cresyl violet staining revealed preserved neuronal morphology in the hippocampal CA1 region and cortex of control animals, characterized by well‐defined neuronal cell bodies and prominent Nissl substance (green arrows). In contrast, ISO‐treated rats exhibited noticeable histoarchitectural alterations, including the presence of shrunken, darkly stained neurons and disrupted cellular organization (red arrows). Treatment with dapagliflozin showed partial improvement in neuronal morphology, with a reduced presence of morphologically altered neurons compared with the ISO group. Notably, the dapagliflozin‐loaded bilosomal formulation further improved neuronal appearance, showing a greater proportion of neurons with preserved morphology and fewer darkly stained cells, although the architecture did not fully resemble that of the control group (Figure 7).

**FIGURE 7:**
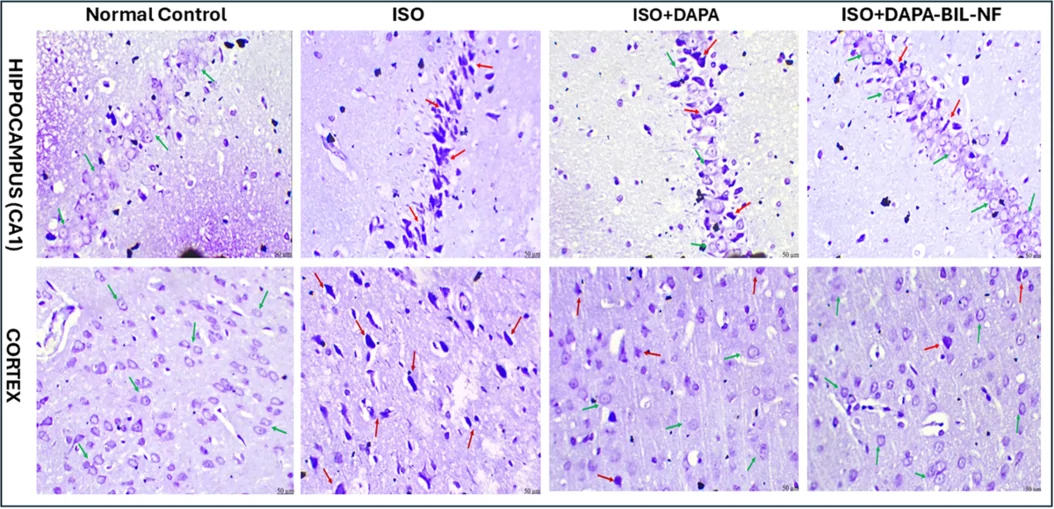
Representative Cresyl violet–stained sections of hippocampal CA1 region and cortex showing neuronal morphology and Nissl substance distribution across experimental groups. Control sections display well‐defined neuronal cell bodies with prominent Nissl substance. ISO‐treated rats show shrunken, darkly stained neurons and disrupted cytoarchitecture. Dapagliflozin treatment partially improves neuronal morphology, whereas DAPA‐BIL‐NF treatment shows a higher proportion of preserved neurons. Scale bar = 50 µm.

### 3.5 Immunohistochemical Analysis of the Hippocampus (CA1 Region) and Cortex

#### 3.5.1 Immunohistochemical Analysis of SIRT6 Expression in the Hippocampus (CA1 Region) and Cortex

Immunohistochemical analysis revealed strong nuclear SIRT6 immunoreactivity in neurons of the hippocampal CA1 region and cerebral cortex of normal control rats (red arrows). In contrast, ISO administration markedly reduced SIRT6 immunoreactivity in both regions, with fewer SIRT6‐positive neurons observed (green arrows). Treatment with dapagliflozin partially restored SIRT6 expression, whereas the dapagliflozin‐loaded bilosomal formulation produced a more pronounced increase in SIRT6 immunoreactivity compared with the ISO group. Quantitative analysis of SIRT6‐positive neurons further confirmed these observations, demonstrating a significant reduction following ISO administration and a significant increase following DAPA and DAPA‐BIL‐NF treatment (Figure 8).

**FIGURE 8:**
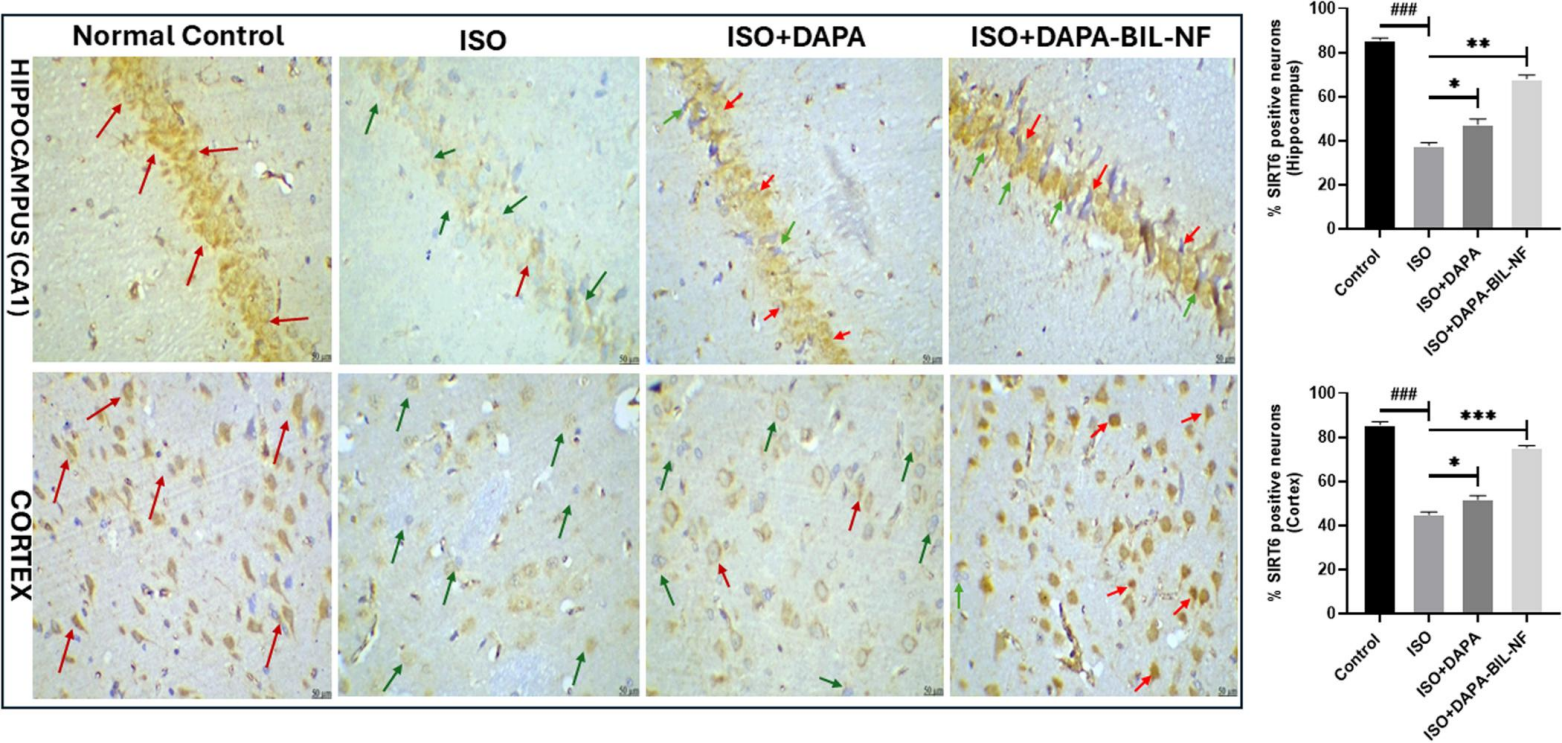
Representative immunohistochemical staining and quantitative analysis of SIRT6 expression in the CA1 region of the hippocampus (upper panel) and cerebral cortex (lower panel) following isoproterenol (ISO) administration (scale bar = 50 μm). Normal control animals exhibited strong nuclear SIRT6 immunoreactivity (red arrows), whereas ISO treatment markedly reduced SIRT6‐positive staining (green arrows). Treatment with dapagliflozin (ISO + DAPA) partially restored SIRT6 expression, while the dapagliflozin‐loaded bilosomal nanovesicular formulation (ISO + DAPA‐BIL‐NF) produced a more pronounced increase in SIRT6 immunoreactivity, approaching normal control levels. Quantitative analysis of SIRT6‐positive neurons is presented as mean ± SEM (*n* = 6). Statistical analysis was performed using one‐way ANOVA followed by Tukey's multiple comparison post hoc test. ^###^ *p* < 0.001 vs. Normal Control (NC); *p < 0.05, **p < 0.01, and *p < 0.001 vs. the ISO group. Red arrows indicate SIRT6‐positive immunostaining, whereas green arrows indicate reduced or weak SIRT6 immunoreactivity.

#### 3.5.2 Immunohistochemical Analysis of TRAP1 Expression in the Hippocampus (CA1 Region) and Cortex

Immunohistochemical analysis revealed strong neuronal TRAP1 immunoreactivity in the hippocampal CA1 region and cerebral cortex of normal control rats (green arrows). In contrast, ISO‐treated animals exhibited markedly reduced TRAP1 immunoreactivity, with a greater number of neurons showing weak or absent staining (red arrows). Treatment with dapagliflozin partially restored TRAP1 expression, whereas the dapagliflozin‐loaded bilosomal formulation produced a more pronounced increase in TRAP1 immunoreactivity compared with the ISO group. Quantitative analysis of TRAP1‐positive neurons further confirmed these observations, demonstrating a significant reduction following ISO administration and a significant increase following DAPA and DAPA‐BIL‐NF treatment (Figure 9).

**FIGURE 9:**
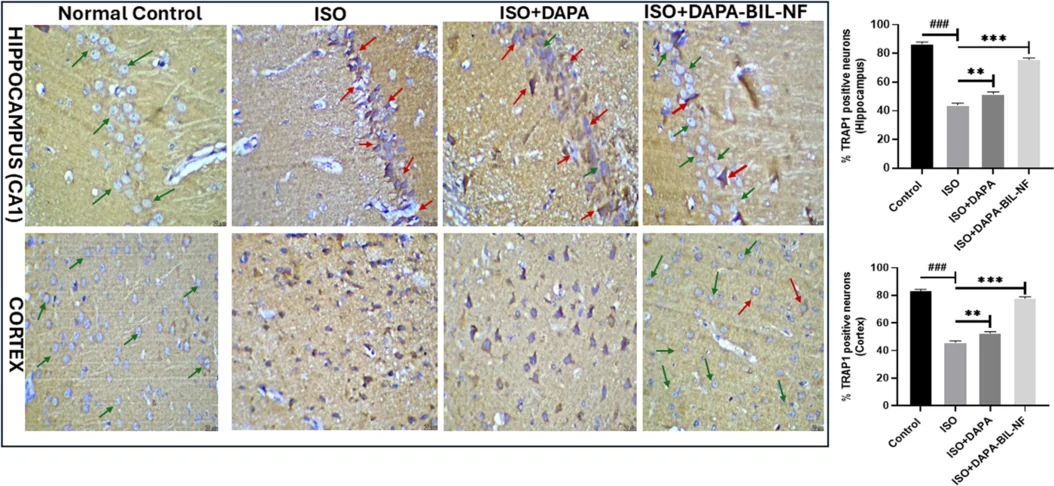
Representative immunohistochemical staining and quantitative analysis of TRAP1 expression in the CA1 region of the hippocampus (upper panel) and cerebral cortex (lower panel) following isoproterenol (ISO) administration (scale bar = 50 μm). Normal control animals exhibited strong neuronal TRAP1 immunoreactivity (green arrows), whereas ISO treatment markedly reduced TRAP1‐positive staining (red arrows). Treatment with dapagliflozin (ISO + DAPA) partially restored TRAP1 expression, while the dapagliflozin‐loaded bilosomal nanovesicular formulation (ISO + DAPA‐BIL‐NF) produced a more pronounced increase in TRAP1 immunoreactivity, approaching normal control levels. Quantitative analysis of TRAP1‐positive neurons is presented as mean ± SEM (*n* = 6). Statistical analysis was performed using one‐way ANOVA followed by Tukey's multiple comparison post hoc test. ^**###**^ *p* < 0.001 vs. Normal Control (NC); **p < 0.01 and ***p < 0.001 vs. the ISO group. Green arrows indicate TRAP1‐positive immunostaining, whereas red arrows indicate reduced or weak TRAP1 immunoreactivity.

#### 3.5.3 Immunohistochemical Analysis of BDNF Expression in the Hippocampus (CA1 Region) and Cortex

Immunohistochemical analysis revealed clear neuronal BDNF immunoreactivity in the hippocampal CA1 region and cortex of control rats (red arrows), whereas ISO treatment markedly reduced BDNF staining in both regions (green arrows). Dapagliflozin treatment partially restored BDNF immunoreactivity, while the dapagliflozin‐loaded bilosomal formulation showed more pronounced neuronal BDNF staining compared with the ISO group (red arrows). Quantitative analysis of BDNF‐positive neurons further confirmed these observations, demonstrating a significant reduction in BDNF expression following ISO administration and a marked increase following DAPA and DAPA‐BIL‐NF treatment (Figure 10).

**FIGURE 10:**
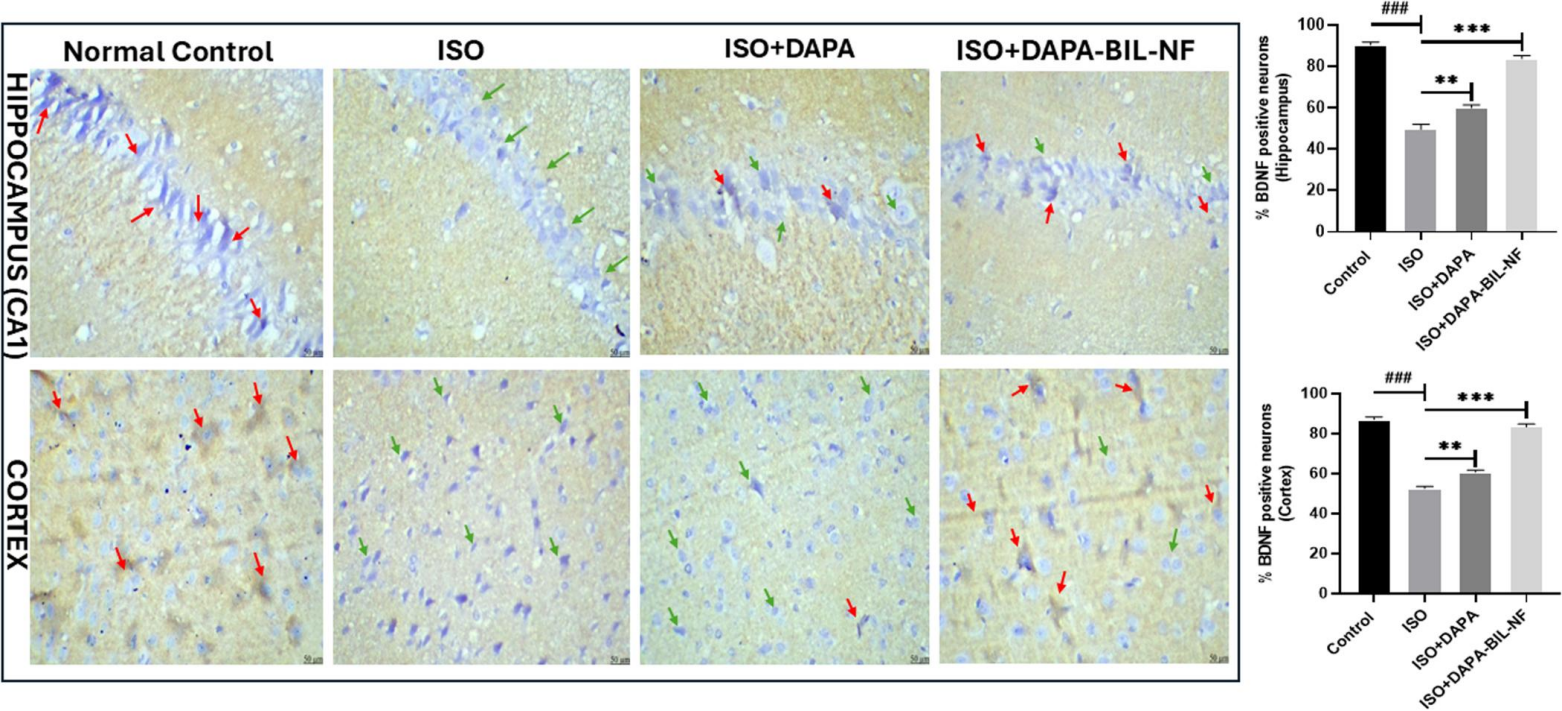
Representative immunohistochemical staining and quantitative analysis of brain‐derived neurotrophic factor (BDNF) expression in the CA1 region of the hippocampus (upper panel) and cerebral cortex (lower panel) following isoproterenol (ISO) administration (scale bar = 50 μm). Normal control animals exhibited strong neuronal BDNF immunoreactivity (red arrows), whereas ISO treatment markedly reduced BDNF‐positive staining (green arrows). Treatment with dapagliflozin (ISO + DAPA) partially restored BDNF expression, while the dapagliflozin‐loaded bilosomal nanovesicular formulation (ISO + DAPA‐BIL‐NF) produced a more pronounced increase in BDNF expression, approaching normal control levels. Quantitative analysis of BDNF‐positive neurons is presented as mean ± SEM (*n* = 6). Statistical analyses were performed using one‐way ANOVA followed by Tukey's multiple comparison post hoc test. ^**###**^ *p* < 0.001 vs. Normal Control (NC); **p < 0.01 and ***p < 0.001 vs. ISO group. Red arrows indicate BDNF‐positive immunostaining, whereas green arrows indicate reduced or weak BDNF immunoreactivity.

#### 3.5.4 Immunohistochemical Analysis of GFAP Expression in the Hippocampus (CA1 Region) and Cortex

Astrocytic GFAP expression was assessed in both the hippocampal CA1 region and the cortex. Minimal GFAP immunoreactivity was observed in control brain sections (green arrows). ISO‐treated rats exhibited intense GFAP staining in hippocampal and cortical regions (red arrows). DAPA treatment partially reduced GFAP expression, while DAPA‐BIL‐NF treatment resulted in marked attenuation of GFAP immunoreactivity compared with the ISO group. Quantitative analysis of GFAP‐positive cells further confirmed these observations, demonstrating a significant increase in GFAP expression following ISO administration and a marked reduction following DAPA and DAPA‐BIL‐NF treatment (Figure 11).

**FIGURE 11:**
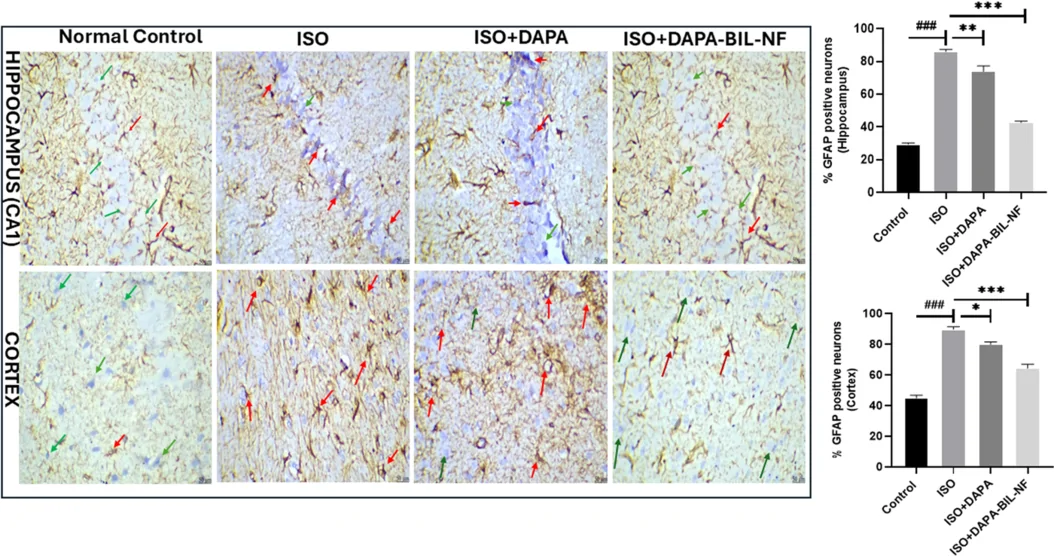
Representative immunohistochemical staining and quantitative analysis of glial fibrillary acidic protein (GFAP) expression in the CA1 region of the hippocampus (upper panel) and cerebral cortex (lower panel) following isoproterenol (ISO) administration (scale bar = 50 μm). Normal control animals exhibited minimal GFAP immunoreactivity (green arrows), whereas ISO treatment markedly increased astrocytic GFAP‐positive staining (red arrows), indicating reactive astrogliosis. Treatment with dapagliflozin (ISO + DAPA) partially attenuated GFAP expression, while the dapagliflozin‐loaded bilosomal nanovesicular formulation (ISO + DAPA‐BIL‐NF) produced a more pronounced reduction in GFAP immunoreactivity. Quantitative analysis of GFAP‐positive cells is presented as mean ± SEM (*n* = 6). Statistical analyses were performed using one‐way ANOVA followed by Tukey's multiple comparison post hoc test. ^**###**^ *p* < 0.001 vs. Normal Control (NC); *p < 0.05, **p < 0.01, and ***p < 0.001 vs. ISO group. Red arrows indicate increased GFAP‐positive astrocytes, whereas green arrows indicate minimal or reduced GFAP immunoreactivity.

## 4 Discussion

Isoproterenol (ISO), a potent β‐adrenergic agonist, is widely employed to induce myocardial injury; however, sustained catecholaminergic stimulation is increasingly recognized to exert systemic toxicity extending to the central nervous system. Excessive adrenergic activation promotes oxidative stress, inflammatory signaling, mitochondrial dysfunction, and progressive neuronal injury [1, 20]. In the present study, ISO administration produced marked biochemical, behavioral, histological, and molecular alterations in the hippocampus and cortex, while dapagliflozin (DAPA), particularly in its bile salt–stabilized nanovesicular formulation (DAPA‐BIL‐NF), significantly mitigated these changes. The enhanced efficacy of DAPA‐BIL‐NF may be attributed to our previously established physicochemical properties, including nanoscale vesicle size, favorable zeta potential, high drug entrapment efficiency, and sustained release characteristics, which collectively facilitate improved drug delivery and tissue exposure [10].

Oxidative stress represents an early and central event in catecholamine‐induced neurotoxicity. Persistent β‐adrenergic stimulation enhances reactive oxygen species generation through NADPH oxidase activation and mitochondrial perturbation, leading to depletion of endogenous antioxidant defences and lipid peroxidation [21, 22]. Consistent with this mechanism, ISO exposure significantly reduced SOD, CAT, and GSH levels while elevating TBARS (Figure 2) [23]. Dapagliflozin restored antioxidant enzyme activity and reduced lipid peroxidation [24], whereas DAPA‐BIL‐NF demonstrated superior normalization of redox parameters, likely reflecting improved tissue exposure and sustained pharmacological engagement. Importantly, neither DAPA alone nor DAPA‐BIL‐NF alone significantly altered oxidative stress markers compared with the normal control group, indicating that the observed antioxidant effects primarily reflect protection against ISO‐induced oxidative injury rather than modulation of basal redox homeostasis.

Oxidative stress and neuroinflammation are tightly interconnected processes [25]. Reactive oxygen species activate redox‐sensitive transcription factors resulting in increased production of pro‐inflammatory cytokines, including TNF‐α, IL‐6, and IL‐1β [26, 27]. In agreement with this cascade, ISO administration significantly elevated pro‐inflammatory mediators and reduced anti‐inflammatory signaling [28] (Figure 3), indicating activation of the oxidative–inflammatory axis. Dapagliflozin attenuated these cytokine alterations, and the nanovesicular formulation exerted a more pronounced suppressive effect, supporting modulation of oxidative–inflammatory crosstalk as a central protective mechanism. Similarly, DAPA alone and DAPA‐BIL‐NF alone did not significantly affect basal TNF‐α, IL‐6, IL‐1β, or IL‐10 levels compared with the Normal Control group, further supporting that the anti‐inflammatory effects observed are specific to pathological conditions induced by ISO.

The biochemical and inflammatory improvements were reflected at the functional level. ISO‐treated animals exhibited anxiety‐like and impaired behavioral performance in the elevated plus maze and forced swim test, respectively (Figures 4 and 5) [29, 30, 31]. Such behavioral impairments are consistent with hippocampal oxidative injury and cytokine‐mediated neuronal dysfunction. Treatment with dapagliflozin improved behavioral performance, and DAPA‐BIL‐NF further enhanced these outcomes, demonstrating that attenuation of molecular injury translated into meaningful functional recovery.

Histopathological examination corroborated these findings. ISO exposure induced neuronal degeneration, pyknosis, and cytoarchitectural disruption in the CA1 region of the hippocampus and cortex [7] (Figure 6). Cresyl violet staining further confirmed loss of neuronal integrity and Nissl substance (Figure 7). Dapagliflozin partially preserved neuronal morphology, whereas DAPA‐BIL‐NF demonstrated improved preservation of tissue architecture.

Beyond oxidative and inflammatory injury, sustained catecholaminergic stress impairs cellular stress‐adaptive pathways essential for neuronal survival. SIRT6 contributes to genomic stability and metabolic–inflammatory regulation [32, 33], whereas the mitochondrial chaperone TRAP1 maintains proteostatic balance and restrains mitochondrial reactive oxygen species accumulation [34, 35]. Together, these stress‐response systems support mitochondrial integrity under conditions of adrenergic overload. ISO significantly reduced SIRT6 and TRAP1 immunoreactivity in hippocampal and cortical regions (Figures 8 and 9), suggesting compromised cellular stress‐response capacity. Dapagliflozin treatment partially restored expression of these proteins, and the nanovesicular formulation produced more robust preservation, indicating improved maintenance of mitochondrial and genomic resilience under adrenergic stress. While the present study employed immunohistochemical evaluation to localize regional protein alterations, future studies incorporating complementary quantitative protein techniques, such as Western blotting or targeted proteomic analyses, would further strengthen these observations.

Markers of neuronal support and glial activation were likewise affected. Reduced brain‐derived neurotrophic factor (BDNF) expression reflects impaired neurotrophic signaling and synaptic plasticity, whereas increased glial fibrillary acidic protein (GFAP) expression denotes astrocytic activation and neuroinflammatory engagement. ISO administration decreased BDNF and increased GFAP expression (Figures 10 and 11), consistent with disrupted neuronal–glial homeostasis [7]. Dapagliflozin partially normalized these markers, and DAPA‐BIL‐NF produced a more pronounced improvement, suggesting preservation of neurotrophic support and attenuation of astrogliosis.

The present study has certain limitations. Although DAPA‐BIL‐NF demonstrated superior efficacy compared with free dapagliflozin, an empty bilosomal vehicle control was not included. However, the inclusion of DAPA‐alone and DAPA‐BIL‐NF‐alone groups demonstrated no significant alterations in basal oxidative stress or inflammatory biomarkers compared with the normal control group, suggesting that the observed biochemical protective effects are unlikely to be attributable to intrinsic effects of the formulation itself. Nevertheless, the possibility of carrier‐related influences on other endpoints cannot be completely excluded. Future studies incorporating an empty nanovesicular control together with pharmacokinetic and biodistribution analyses would further clarify the individual contribution of the carrier system.

Collectively, the findings delineate a mechanistic cascade in which sustained adrenergic stimulation induces oxidative stress, activates inflammatory signaling, disrupts mitochondrial and stress‐response proteins, impairs neurotrophic support, and culminates in behavioral dysfunction and structural degeneration. Dapagliflozin attenuates multiple components of this cascade, and the enhanced efficacy observed with the nanovesicular formulation most plausibly reflects improved pharmacokinetic delivery and tissue exposure rather than activation of a novel molecular pathway. These results underscore the therapeutic relevance of targeting oxidative–inflammatory neurotoxicity in catecholamine‐mediated brain injury and support formulation‐based strategies to optimize neuroprotective interventions under sustained adrenergic stress.

## 5 Conclusion

The present study demonstrates that dapagliflozin attenuates isoproterenol‐induced catecholaminergic brain injury, as evidenced by attenuation of oxidative stress and neuroinflammation, preservation of stress‐responsive and neurotrophic protein expression, improvement in behavioral outcomes, and maintenance of neuronal architecture in hippocampal and cortical regions. Delivery of dapagliflozin via a bile salt–stabilized nanovesicular system resulted in more pronounced normalization of these injury‐associated alterations compared with the free drug, consistent with formulation‐assisted enhancement of pharmacological efficacy. Rather than indicating engagement of a distinct signaling pathway, these findings reflect coordinated modulation of established oxidative–inflammatory and cellular stress‐response mechanisms under sustained β‐adrenergic stress. Collectively, this work supports the therapeutic potential of formulation‐based strategies to improve the efficacy of established pharmacological agents in conditions associated with chronic catecholaminergic injury.

## Author Contributions

**Faisal Ashraf Bhat:** conceptualization, methodology, funding acquisition, validation, writing – original draft, data curation, software, writing – review and editing. **Syed Ehtaishamul Haque:** investigation, validation, visualization. **Mohammed Aqil:** validation, investigation, formal analysis. **Mohd Akhtar:** formal analysis, visualization. **Abul Kalam Najmi:** conceptualization, project administration, supervision, data curation, resources, funding acquisition, investigation, writing – review and editing, validation, methodology.

## Consent

The authors declare that they have provided their consent to publish this manuscript.

## Conflicts of Interest

The authors declare no conflicts of interest.

## Data Availability

The data that support the findings of this study are available from the corresponding author upon reasonable request.
